# Does the plasticity of neural stem cells and neurogenesis make them biosensors of disease and damage?

**DOI:** 10.3389/fnins.2022.977209

**Published:** 2022-09-08

**Authors:** Ane Rodríguez-Bodero, Juan Manuel Encinas-Pérez

**Affiliations:** ^1^The Neural Stem Cells and Neurogenesis Lab, Achucarro Basque Center for Neuroscience, Leioa, Spain; ^2^Department of Neuroscience, University of the Basque Country (UPV/EHU), Leioa, Spain; ^3^IKERBASQUE, The Basque Foundation for Science, Bilbao, Spain

**Keywords:** adult neurogenesis, hippocampus, neural stem cells, aberrant neurogenesis, neuroinflammation, neuronal hyperexcitation

## Abstract

Postnatal and adult neurogenesis takes place in the dentate gyrus of the hippocampus in the vast majority of mammals due to the persistence of a population of neural stem cells (NSCs) that also generate astrocytes and more NSCs. These are highly plastic and dynamic phenomena that undergo continuous modifications in response to the changes brain homeostasis. The properties of NSCs as well as the process of neurogenesis and gliogenesis, are reshaped divergently by changes in neuronal activity and by different types of disease and damage. This richness of plastic responses identifies NSCs and newborn neurons as biosensors of the health state of the hippocampus, detecting and providing useful information about processes such as neuronal and network hyperexcitation, excitotoxicity, neurodegeneration, and neuroinflammation. Learning to gather and use this information is a challenge worth of our attention.

## Plasticity and hippocampal neurogenesis

A fascinating capability of the mammalian brain is its plasticity. The interaction with the environment triggers patterns of neural activity that in turn modify the activity of neurons and neural networks, altering perception and behavior. The hippocampus is a brain structure particularly rich in plasticity as one of its main functions is to convert experiences into memory traces which help optimize behavior, a process called learning. In the hippocampus, synaptic plasticity, consisting in the potentiation or depression of the efficacy of synaptic transmission, is accompanied by another type of plasticity: neurogenesis, the generation of new neurons from neural stem cells (NSCs). The process of neurogenesis continuously reacts in a variety of manners to changes in brain homeostasis, acting as a sensor that provides information about ongoing processes such as neuronal hyperexcitation or neuroinflammation.

Between the hilus and the granule cell layer (GCL) of the dentate gyrus (DG), in a narrow and loose area called the subgranular zone (SGZ) a population of neural stem cells (NSCs) keeps generating neurons throughout adult life of most mammals. Mounting evidence suggest that postnatal and adult hippocampal neurogenesis also take place in the human brain (Terreros-Roncal et al., 2021), as others forms of plasticity do (reviewed in Bonfanti and Charvet, 2021), although further work is necessary to fully characterize its features and extent.

**FIGURE 1:**
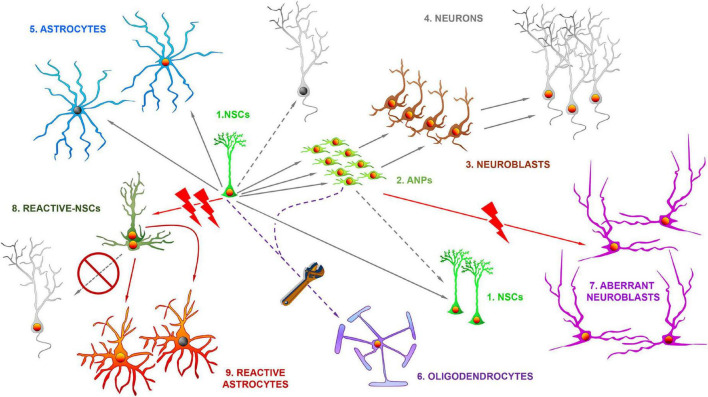
The differential fate of multipotent adult hippocampal neural stem cells in health and disease. In normal conditions, the hippocampal neural stem cells (NSCs, 1) of the dentate gyrus get activated at a low rate and divide asymmetrically to generate amplifying neural progenitors (ANPs, 2) which ultimately differentiate into immature neurons (neuroblasts, 3) and ultimately in fully mature neurons (4) of the granule cell layer. The direct differentiation into neurons is also possible. NSCs also generate astrocytes (5) through ultimate differentiation. NSCs can also divide symmetrically to generate copies of themselves. By gene expression manipulation NSCs can also differentiate into oligodendrocytes (6). In situations of pathological neuronal hyperexcitation NSCs such as epilepsy, can generate aberrant neuroblasts (7), characterized by abnormal migration, dendritic arborization and dendritic spine density. In more extreme cases such as temporal lobe epilepsy with hippocampal sclerosis, NSCs abandon neurogenesis and transform into reactive-NSCs (8), which abandon neurogenesis, get activated massively to enter symmetric cell division and ultimately differentiate into reactive astrocytes (9).

Hippocampal NSCs with astrocytic properties were shown to give rise to neurons (Seri et al., 2001; Figure 1) and for several years this was the only lineage fate attributed to them. However, gliogenesis takes place in parallel to neurogenesis in the DG (Steiner et al., 2004) and it was later confirmed that NSCs could differentiate into astrocytes after getting activated to generate neuronal progenitors (Encinas et al., 2011). NSCs were also found to be able to enter symmetric division to generate copies of themselves (Bonaguidi et al., 2011; Pilz et al., 2018; Figure 1). Further, hippocampal NSCs could also generate oligodendrocytes, at the expense of neurogenesis, after gene manipulation (Jessberger et al., 2008). Symmetric division could potentially compensate the natural depletion of NSCs that is concomitant to their activation to generate neuronal progenitors, and that takes place through direct differentiation into astrocytes or neurons (Bonaguidi et al., 2011; Encinas et al., 2011; Pilz et al., 2018). However, symmetric division in normal conditions is just not abundant enough to compensate depletion. Symmetric divisions of NSCs can, however, be transiently induced through gene expression manipulation to repopulate the niche and increase the level of neurogenesis in the aged hippocampus (Berdugo-Vega et al., 2020). A natural manner of preventing the depletion of NSCs is by putting a brake to their activation. The immediate consequence would be reduced neurogenesis, but on the other hand, albeit at lower level, it could be maintained for a much longer period. Indeed, at least in rodents, a model of fast spending (higher NSC activation and faster depletion) transitions into a model of conservative saving (lower activation and slower depletion) (Encinas et al., 2011; Martín-Suárez et al., 2019).

Several molecular mechanisms have been recently identified that regulate this transition (discussed in Martín-Suárez and Encinas, 2021), and one of them, neuroinflammation, as explained later, can be key in regulating NSCs in disease and damage. Overall, these findings show how plastic NSCs and neurogenesis can be in normal conditions. A final point to consider is that, as beautifully proposed in another article of these series (Urbán, 2022), the different pathways and fates of NSCs should not be considered as isolated irreversible states, but rather as a continuum of possibilities whose probability of materialization increases or decreases depending on intrinsic properties and external cues, including those generated by disease and damage. Finally, adding another layer of complexity, individual variations in the amount of neurogenesis correlate with individual behavioral differences (Freund et al., 2013). This neurogenic individuality could be also supported by other variables such as number of NSCs, rate of activation, connectivity of newborn neurons… and of course how they are modified by damage and disease.

## Neural stem cells as biosensors

### Inactivation of neural stem cells

**FIGURE 2:**
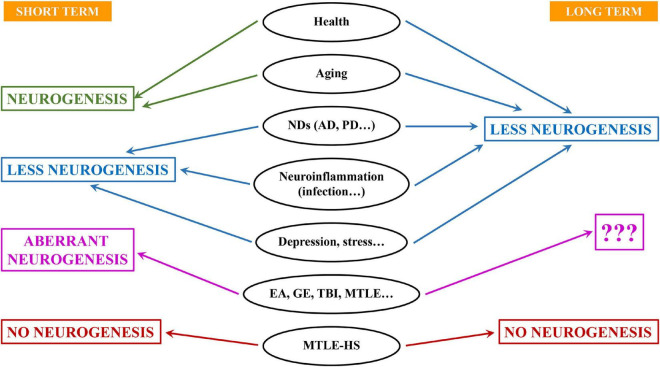
Different outcomes for neural stem cells and neurogenesis in aging, disease and damage. Aging, neurodegeneration and infection are accompanied by neuroinflammation and typically associate with lower levels of neurogenesis at least in the long term. On the contrary, situations of neuronal hyperexcitation (EA, epileptiform activity; GE, generalized epilepsy; TBI, traumatic brain injury; MTLE, mesial temporal lobe epilepsy) usually trigger an increase of neurogenesis in the short term. However, neurons born under these conditions are aberrant. It is not clear whether this effect is stained in long-term. In more severe forms of epilepsy such as MTLE with hippocampal sclerosis (MTLE-HS), neurogenesis is drastically impaired even in the short term.

Cell proliferation, NSC activation and production of newborn neurons are markedly reduced by interleukins, interferons and tumor necrosis factor-α (reviewed in Das and Basu, 2008; Figure 2) and neurogenesis can be restored by non-steroidal anti-inflammatory drugs such as indomethacin or minocycline (Ekdahl et al., 2003; Monje et al., 2003). Further, neuroinflammation could be the principal actor streaming together neurodegeneration, bacterial and viral infection and aging to impair NSC activity. A very interesting aspect of the transition between the fast spending to the conservative saving model that takes place over time is that is based on the progressive deepening of NSCs quiescence. Early in adulthood the population of NSCs commences to diverge into at least two subpopulations. One that keeps behaving in terms of activation and neuron generation without major changes, and another population whose probability of activation falls (reviewed in Martín-Suárez and Encinas, 2021). Several factors have been identified to cause the deepening in quiescence of NSCs: increasing expression of glucocorticoid receptor (Schouten et al., 2020); the interplay between ASCL1 (Achaete-Scute Family BHLH Transcription Factor 1) and the E3 ubiquitin ligase HUWE1 (Harris et al., 2021); the interplay between nuclear lamina protein lamin B1 and SUN-domain containing protein 1 (SUN1) (Bin Imtiaz et al., 2021) and tyrosine-protein kinase Abl1 (Ibrayeva et al., 2021). Interestingly another key factor is neuroinflammation. In the hippocampus (Martín-Suárez et al., 2019) as well as in the subventricular zone (Kalamakis et al., 2019), increased interferons signaling promote quiescence of NSCs. Further, the level of reactive oxygen species, key factors in inflammatory processes, is linked to the equilibrium between quiescence and activation of NSCs (Adusumilli et al., 2021). Are all these mechanisms shared by the conditions in which neuroinflammation is triggered?

### Overactivation of neural stem cells

Increased neuronal activity increase NSCs activation, with GABAergic receptors playing an important role (Ge et al., 2007). This could be an important mechanism in situations of pathological neuronal hyperexcitation such as epilepsy, in which GABAergic transmission is severely impaired. The hippocampus is more vulnerable to neuronal hyperexcitation and excitotoxicity than other brain structures. In addition, because of its recurrent circuits, it is frequently the origin of seizures in epilepsy. Mesial temporal lobe epilepsy (MTLE) is the most common form of epilepsy and a main characteristic is that the hippocampus, or sometimes related structures such as the amygdala, are the focus of seizures (Wieser and ILAE Commission on Neurosurgery of Epilepsy, 2004). Thus, studying NSCs in neurogenesis in MTLE has gained increased attention.

In experimental models of neuronal hyperexcitation, overactivation of NSCs has been consistently reported, using experimental electroshock (Segi-Nishida et al., 2008) or the induction of seizures (Hüttmann et al., 2003; Sierra et al., 2015). An obvious prediction is that if neural hyperexcitation is maintained, NSCs overactivation would provide a boost of neurogenesis, but depletion linked to activation (Encinas et al., 2011; Pilz et al., 2018) would deplete the NSCs pool and impair neurogenesis in the long run. An intriguing possibility is that symmetric division was also increased and thus the pool of NSCs could be maintained and neurogenesis kept at higher levels for longer periods. The scenario confirmed so far is the chronic impairment of neurogenesis due to accelerated depletion of NSCs (Sierra et al., 2015) and could explain recent findings that suggest that neurogenesis decreases faster over time in the hippocampus of epilepsy patients (Ammothumkandy et al., 2022).

Interestingly, in some cases of higher neuronal hyperexcitation, NSCs not only can get activated massively, but can switch their behavior in a dramatic manner. Around one third of MTLE patients do not respond to drugs and unilateral hippocampalectomy is the last therapeutic resort of choice to control seizures (Wieser and ILAE Commission on Neurosurgery of Epilepsy, 2004). These patients typically present MTLE with hippocampal sclerosis (MTLE-HS) which is characterized by unilateral neuronal loss in CA, GCL dispersion and intense reactive gliosis and neuroinflammation. In mouse models of MTLE-HS, NSCs get activated massively as they become hypertrophic, multibranched and migrate away from the SGZ. Importantly, these reactive NSCs (React-NSCs) enter symmetric division and neurogenesis is abandoned (Sierra et al., 2015; Muro-García et al., 2019). During a few weeks React-NSCs are different from NSCs, astrocytes and reactive astrocytes (Valcárcel-Martín et al., 2020), but they ultimately differentiate into reactive astrocytes. Their functional contribution to gliosis is still ignored. In general, reactive gliosis associates with neuroinflammation which depending on the type of insult or disease and the timing can be detrimental or beneficial. In the particular case of epilepsy reactive gliosis is considered to be epileptogenic for several reasons: potassium buffering and GABA synthesis are compromised, cytokines modify neuronal activity, etc. (Abbracchio et al., 2009). Besides the inevitable loss of local endogenous regenerative potential and loss of the cognitive functions associated with neurogenesis, it is intriguing to know what is the contribution of NSCs to epileptogenesis via reactive gliosis. Another question of interest arises then. Even if we are able to prevent the conversion of NSCs into React-NSCs in a situation of reactive gliosis, would they be able to generate healthy neurons? In a situation such as MTLE-HS, the neurogenic niche could be so disrupted that neurogenesis becomes impermissible.

## Newborn neurons as biosensors

### Dystrophic newborn neurons

**FIGURE 3:**
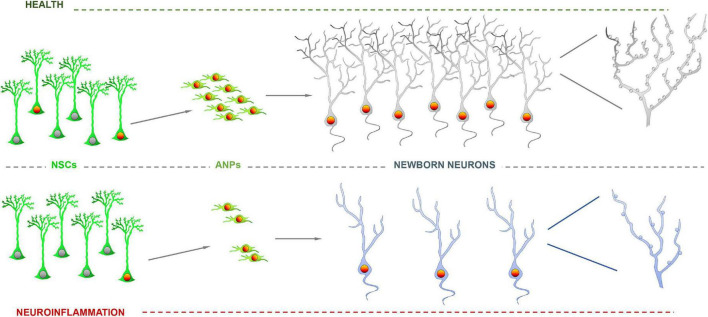
Impairment of neurogenesis due to neuroinflammation. Neural stem cell (NSCs) activation and precursor proliferation are reduced by inflammatory agents such as interleukins, which translates in a lower number of newborn neurons. Further, neurons born or differentiated under neuroinflammation exhibit a dystrophic phenotype with less developed dendritic arborization and fewer dendritic spines.

Indeed, newborn neurons, even in immature states as progenitors and neuroblasts, are also highly sensible to changes in the niche and can respond in different manners. Typically, neuroinflammation, without, provokes a reduction in cell proliferation (NSCs and precursors) but also the alteration of newborn neurons whose maturation, in terms of dendritic arborization and synaptic connections is reduced (Valero et al., 2014) and present a dystrophic phenotype. These effects are very similar to those reported for models of neurodegenerative disorders such as Alzheimer’s Disease (Valero et al., 2014; Wang et al., 2016; Figure 2) and could be the case too for neurons born in the human hippocampus (Terreros-Roncal et al., 2021). This finding should not come as a surprise, as neurodegeneration and neuroinflammation are tightly connected. In this case too, loss of GABAergic transmission has also been shown to impair neurogenesis in AD models (Wang et al., 2014), most likely as a direct effect as GABAergic transmission also regulates the differentiation and synaptic integration of newborn neurons (Ge et al., 2006). Dystrophic morphology can be interpreted as diminished maturation when dendrites are less elaborated and the density on dendritic spines is lower, as it is the case in models of neuroinflammation-neurodegeneration (Valero et al., 2014; Wang et al., 2016). But the opposite effect, more developed dendritic arborization richer in dendritic spines has also been found, for instance in a model of glucocorticoid administration (Schouten et al., 2020). Nevertheless, this effect could be due not just to the level of glucocorticoids, but to the abolition of its oscillations. Selective knock-down of glucocorticoid receptors in newborn neurons also triggers an increase in dendritic arborization and higher density of dendritic spines (Fitzsimons et al., 2013). Glucocorticoids, main mediators in stress, reduce NSCs and neuronal precursor proliferation as neuroinflammation does (Figure 3). But as we have just seen, this effect does not necessarily correlate with delayed or advanced differentiation. What is the functional electrophysiological relevance of a dystrophic morphology? This aspect has been much less addressed, but when addressed, modified morphology changes were found to be accompanied by functional changes (Duan et al., 2007; Fitzsimons et al., 2013; Kerloch et al., 2021). Several molecular mechanisms have been found to regulate the differentiation, at least in terms of morphological development of newborn neurons, such as disrupted-in-schizophrenia-1 (Duan et al., 2007); Rho GTPase Rnd2 (Kerloch et al., 2021) and glycogen synthase kinase-3β glycogen synthase kinase-3β (Llorens-Martín et al., 2013). What is the role of these mechanisms, and what others could be involved, in disease-induced newborn neuron dystrophy remains to be fully explored. An aspect that is usually overlooked is whether these alterations are transient, lasting or even permanent.

### Aberrant newborn neurons

Conditions with neuronal hyperexcitation as an essential component deserve to be commented separately as there is one key factor in the neurogenic response. In opposition to the situations that we just discussed, neuronal hyperexcitation typically markedly increases neurogenesis, except, that is, in the extreme case of MTLE-HS. Pioneering work on an experimental model of MTLE showed for the first time that seizure triggered a sharp increase in neurogenesis (Parent et al., 1998), but importantly, that newborn neurons presented noticeable abnormalities such as abnormal migration and abnormal dendritic arborization with frequent basal and lateral dendrites in addition to altered main apical dendrite. Together these alterations were termed as aberrant neurogenesis (Parent et al., 1997, 2006; Figure 1) and the relevance of this finding lies in the capacity of aberrant newborn neurons to contribute to epileptogenesis during a critical period of their maturation process (Varma et al., 2019; Lybrand et al., 2021). This is an important finding because it showcases how neurogenesis could be directly contributing to pathology as it does not only participate in the promotion of seizures but also in the associated cognitive decline (Cho et al., 2015).

In a mouse model of Dravet Syndrome, a rare but devastating pediatric generalized epilepsy, markedly increased aberrant neurogenesis has also been found (Martín-Suárez et al., 2020) as well as in experimental traumatic brain injury (Ibrahim et al., 2016). Interestingly, a small percentage of TBI patients develop seizures over time. Whether aberrant neurogenesis contribute to seizure generation in DS or TBI is an intriguing possibility that has not been tested. We do not know whether increased aberrant neurogenesis is sustained for long periods, but the data suggest that it is not the case either in experimental models (Jessberger et al., 2007; Sierra et al., 2015) or in human samples (Mathern et al., 2002; Hattiangady et al., 2004). As we commented before, the level of neurogenesis and quality of neurogenesis can be regulated independently and aberrant neurogenesis can be induced in parallel to boosted or decreased neurogenesis (Muro-García et al., 2019).

## Questions and future directions

We have seen that NSCs and neurogenesis can be differentially affected by pathophysiological conditions. NSCs can be underactivated or be overactivated. They can even differentiate into reactive astrocytes. We dare to anticipate that new extra-neurogenic functions of NSCs will be discovered in the near future and that novel capabilities could be of special importance in situations of damage and disease. Newborn neurons can be generated in larger numbers or in lower numbers. Their migration, dendritic arborization and synaptic connectivity can be disrupted, together or independently. All these modifications can also be different in the short term and the long term (Figure 3). The complete range of potential combinatory outcomes is thus astonishingly wide. However, it represents also an opportunity to harvest useful information about the state of the niche and the undergoing processes during disease or after insult. We should first answer several key questions. What is the actual impact of dystrophic or aberrant neurogenesis in the hippocampal and brain circuitry? Does chronic neuroinflammation related to disease cause a similar effect as the aging-related increase in inflammation (inflammaging)? In other words, does neuroinflammation induced-quiescence deepen over-time? Is there a turning point in which deepened quiescence becomes irreversible senescent-like state? Further, is gliogenesis or self-renewal altered by neuroinflammation? Finally, the key question that triggered the writing of this Perspective is whether we should consider neurogenesis as another readout to better discriminate pathology and how useful it could be as a diagnosis tool. Mounting evidence suggest not only that there is continuous neurogenesis in the human dentate gyrus, but also that as found in mouse models, neurogenesis responds differently in diverse pathologies, acting as biosensor of the undergoing pathological process (Klein et al., 2021; Terreros-Roncal et al., 2021; Ammothumkandy et al., 2022). The main challenge is of course, how to monitor neurogenesis, *in vivo*.

## Data availability statement

The original contributions presented in this study are included in the article/supplementary material, further inquiries can be directed to the corresponding author.

## Author contributions

JME-P conceived and co-wrote the manuscript. AR-B co-wrote the manuscript. Both authors contributed to the article and approved the submitted version.
